# Correction: Effect of pomelo seed-derived carbon on the performance of supercapacitors

**DOI:** 10.1039/d4na90016b

**Published:** 2024-02-01

**Authors:** Zhenyao Yin, Yaping Xu, Jinggao Wu, Jing Huang

**Affiliations:** a State Key Laboratory of Silkworm Genome Biology, Key Laboratory of Sericultural Biology and Genetic Breeding, Ministry of Agriculture and Rural Affairs, College of Sericulture, Textile and Biomass Sciences, Southwest University Chongqing 400715 P. R. China hj41012@163.com; b Institute for Clean Energy & Advanced Materials, Faculty of Materials and Energy, Southwest University Chongqing 400715 P. R. China; c Key Laboratory of Rare Earth Optoelectronic Materials & Devices, College of Chemistry and Materials Engineering, Huaihua University Huaihua 418000 P. R. China

## Abstract

Correction for ‘Effect of pomelo seed-derived carbon on the performance of supercapacitors’ by Zhenyao Yin *et al.*, *Nanoscale Adv.*, 2021,**3**, 2007–2016, DOI: https://doi.org/10.1039/D0NA00778A.

*Nanoscale Advances* is issuing this correction to notify readers that there are portions of text overlap with a number of different sources, the text should have been rewritten to avoid the overlapping text. In addition, the authors regret that some relevant citations to previous work were not included in the original reference list of the published article.

Ref. 24 in the article should be corrected to also include ref. 1 below.

Ref. 29 in the article should be corrected to also include ref. 2 below.

Ref. 37 in the article should be corrected to also include ref. 3 below.

Ref. 38 in the article should be corrected to also include ref. 4 below.

Ref. 44 in the article should be corrected to also include ref. 5 below.

Ref. 50 in the article should be corrected to also include ref. 6 below.

Ref. 52 in the article should be corrected to also include ref. 7 below.

Ref. 64 in the article should be corrected to also include ref. 8 below.

Fig. 3c should be replaced with the following image:
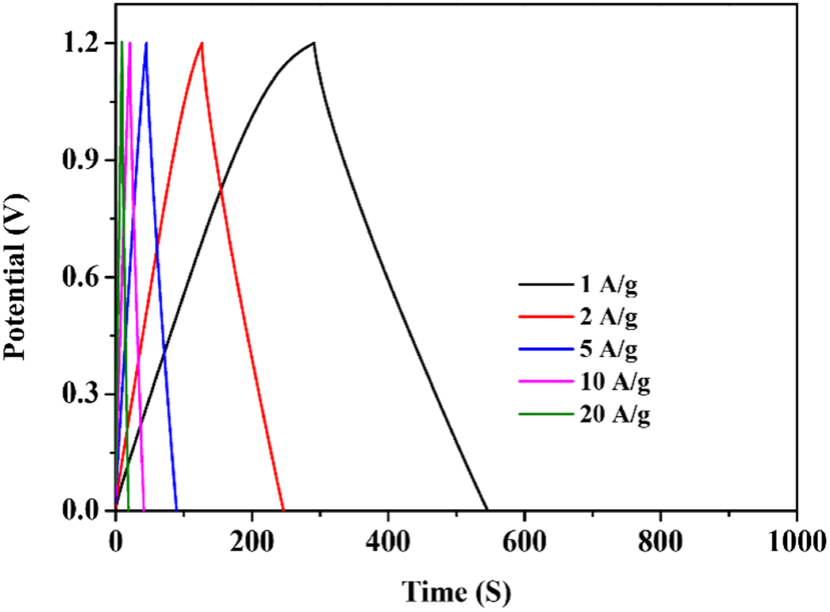


The Royal Society of Chemistry apologises for these errors and any consequent inconvenience to authors and readers.

## Supplementary Material

## References

[cit1] Shi W., Chang B., Yin H., Zhang S., Yang B., Dong X. (2019). Sustainable Energy Fuels.

[cit2] Zhang Q., Han K., Li S., Li M., Li J., Ren K. (2018). Nanoscale.

[cit3] Liu Y., Huang B., Lin X., Xie Z. (2017). J. Mater. Chem. A.

[cit4] Thangavel R., Kannan A. G., Ponraj R., Thangavel V., Kim D. W., Lee Y. S. (2018). J. Mater. Chem. A.

[cit5] Wei L., Sevilla M., Fuertes A. B., Mokaya R., Yushin G. (2011). Adv. Energy Mater..

[cit6] Huang J., Chen J., Yin Z. Y., Wu J. G. (2020). Nanoscale Adv..

[cit7] Qian W., Sun F., Xu Y., Qiu L., Liu C., Wang S., Yan F. (2014). Energy Environ. Sci..

[cit8] Li Y., Zhang D., He J., Wang Y., Zhang X., Zhang Y., Liu X., Wang K., Wang Y. (2019). Sustainable Energy Fuels.

